# Feeling Happy and Sad at the Same Time? Subcultural Differences in Experiencing Mixed Emotions between Han Chinese and Mongolian Chinese

**DOI:** 10.3389/fpsyg.2016.01692

**Published:** 2016-10-27

**Authors:** Xinmei Deng, Xuechen Ding, Chen Cheng, Hiu Mei Chou

**Affiliations:** ^1^College of Psychology and Sociology, Shenzhen UniversityShenzhen, China; ^2^East China Normal UniversityShanghai, China; ^3^Department of Psychology, University of Massachusetts Boston, BostonMA, USA

**Keywords:** mixed emotions, implicit attitudes toward happiness, regulatory motivation, subcultural differences, Implicit Association Test (IAT)

## Abstract

Sometimes people experience pleasant and unpleasant emotions at the same time in a single emotional event. Previous cross-cultural studies indicated that such mixed emotions are more prevalent in China and related to the attitudes toward happiness and individual’s regulatory motivation. However, China is a multi-ethnic country and not much is known about subcultural differences in mixed emotions. The aim of this study was to examine the role that implicit attitudes toward happiness and regulatory motivation played in regard of the subcultural differences in mixed emotions between Han (*N* = 61) and Mongolian Chinese (*N* = 46). Results indicated that, compared with Mongolian Chinese, Han Chinese showed stronger associations between implicit contra-hedonic attitudes toward happiness and mixed emotions during pleasant emotional events. Also, Han Chinese who reported contra-hedonic motivation during pleasant emotional events had higher levels of mixed emotions than those who had hedonic motivation. No significant differences were found in terms of mixed emotions between Mongolian Chinese who had contra-hedonic and hedonic motivation. These results suggest that the psychological mechanisms underlying differences in mixed emotions also require a more comprehensive understanding from a subcultural perspective.

## Introduction

In the bipolar model of affect, unpleasant emotions are generally considered as the opposite of pleasant emotions (Larsen and Mcgraw, 2011). An individual’s emotion experience is assumed to be in a single discrete state arising from a specific emotional event (Barrett et al., 2007). For instance, people generally experience only pleasant emotions when involved in a pleasant emotional event. However, an increasing number of cross-cultural studies indicated that people can experience pleasant and unpleasant emotions at the same time in various occasions (e.g., Larsen et al., 2001; Spencer-Rodgers et al., 2010). For example, graduating from college could make people feel happy for the graduation and sad for leaving college friends. Happiness and sadness occur systematically with mixed affective cues (Hunter et al., 2008). Of note, such mixed emotions are more prevalent in East Asians (e.g., China, Spencer-Rodgers et al., 2010). Prior research indicated that both attitudes toward happiness and regulatory motivation could play a significant role in people’ emotional experiences (Spencer-Rodgers et al., 2010; Miyamoto and Ma, 2011). Thus, we attempted to explore how these two important factors influence people’s emotions in a pleasant emotional event across two different subcultures.

### Contra-Hedonic Attitudes toward Happiness and its Consequence of Mixed Emotions in Cross-Cultural Studies

Attitudes toward emotions refer to evaluations and representations toward different emotions (Riediger et al., 2014). It determines how people evaluate specific emotions and influences what people prefer to experience (Larsen and Mcgraw, 2011). Whether people view specific emotions as desirable highly depend on their cultural model (Mesquita et al., 2014). For example, happiness is defined as the favorable internal feelings and is highly related to individuals’ well-being (Oishi et al., 2013). However, in many occasions, what is considered happy in one culture may be contemplated as neutral or even negative in other cultures (Miyamoto and Ma, 2011). Moreover, prior research indicated that cultural differences are more salient in pleasant emotions in contrast to unpleasant ones (Leu et al., 2011). This kind of contra-hedonic attitudes toward happiness may account for the cultural differences in mixed emotions (Spencer-Rodgers et al., 2010). For instance, in European American culture, which is hedonist at core, individuals are encouraged to pursue the greatest pleasant experience (Uchida and Kitayama, 2009). However, findings from cross-cultural studies suggested that happiness can be potentially dangerous or obstructive in the East Asian cultures (Miyamoto et al., 2010). In the traditional Chinese society, *Yin Yang* philosophy is highly emphasized and encouraged, thus giving rise to a changing philosophy of emotions (e.g., extreme joy begets sorrow, Miyamoto et al., 2014). Larsen et al. (2001) found a strong negative correlation between self-reported pleasant and unpleasant emotions in Americans. On the contrary, Napa Scollon et al. (2005) found that pleasant and unpleasant mood were positively correlated among Asian Americans and Japanese, but not correlated among other groups. Spencer-Rodgers et al. (2010) reported a positive correlation between pleasant and unpleasant emotions in Chinese, supporting the account of dialectical attitudes toward happiness. Also, Hamamura et al. (2008) suggested that dialectical thinkers tolerate more contradictions and counter-positions. Since Chinese are considered to show more dialectical attitudes toward happiness (Spencer-Rodgers et al., 2010), it would not be difficult to understand why they report more mixed emotions than their Western counterparts.

### Contra-Hedonic Regulatory Motivation and its Consequence of Mixed Emotions in Cross-Cultural Studies

Another important factor influencing mixed emotions is regulatory motivation. Regulatory motivation refers to how people regulate their emotions in a specific emotional event (Miyamoto et al., 2014). It includes not only broad classes of desired emotional outcomes (e.g., to feel good) but also instrumental goals (Tamir, 2016). Prior research on emotion regulation demonstrated that cultural goals shape what people want to feel and regulate (Mesquita et al., 2014). For example, contra-hedonic motivation is more prevalent in East Asians cultures (Miyamoto and Ma, 2011). A possible cause is that people prefer to regulate the emotions that facilitate cultural salient tasks (Tamir, 2016). Contra-hedonic motivation is defined as the tendency to enhance unpleasant emotions and suppress pleasant emotions. It is considered to be beneficial to emotional outcomes personally, and it is consistent with the core regulation goal of maintaining interpersonal harmony nationally (e.g., Deng et al., 2013). Mixed emotions could be the experiential consequence of contra-hedonic motivation (Miyamoto et al., 2014).

Although contra-hedonic motivation may not be useful for fulfilling one’s hedonic needs, it may serve utilitarian purpose (Tamir et al., 2007). For example, people who have higher emotional intelligence prefer to make them angry in an interpersonal confronting condition (Ford and Tamir, 2012). When pleasant emotions are considered to be potentially dangerous and unpleasant emotions are beneficial, hedonic cost may be involved even in a pleasant emotional event (Mauss et al., 2011). Such contra-hedonic motivation is more common among Chinese population. For instance, Deng et al. (2013) found that, in a pleasant emotional event, Chinese adolescents who intentionally sacrificed their short-term hedonic needs would experience higher levels of pleasant emotions in a long-term time scale. However, some ethnic minorities in China, such as Mongolian Chinese, despite sharing a collective and interdependent culture with Han Chinese, allow and encourage the expression of happiness in front of others (Li et al., 2012).

### Subcultural Differences in China

Prior cross-cultural studies have shown that individuals in different cultures differ in experiencing mixed emotions (e.g., Chinese vs. Americans, Spencer-Rodgers et al., 2010; Japanese vs. Americans, Miyamoto et al., 2010). Growing evidence demonstrates that subcultural differences also play an important role in people’s daily lives both in multi-ethnic and multi-culture contexts (Cheung et al., 2003; Roberts et al., 2008; Butler et al., 2009). People in diverse social ecologies show variability in psychological orientations, even within a single country (Uskul et al., 2008). According to Eco-cultural theory (Greenfield, 2014), ecological and sociodemographic differences within countries can produce variable cultural values and subcultural characteristics (Uskul et al., 2008). Culture can be conceptualized in local ecology and sociocultural practices, rather than across different countries (Esteban-Guitart et al., 2015). For example, the association between self-esteem and happiness was stronger among Japanese living in relationally mobile regions than among Japanese living in less mobile regions (Yuki et al., 2013). Also, a large number of investigations of American population have shown that people in different subcultures may differ in their emotional experience (Lee et al., 2006), emotion regulation (Boiger et al., 2012), and attitudes toward happiness (Miyamoto et al., 2014). In comparison to European Americans, Asian Americans typically report higher levels of social anxiety, a need to adjust their emotions more frequently, and are more likely to endorse dialectical attitudes toward pleasant emotions. However, to our knowledge, there has been no research conducted on the subcultural differences in mixed emotions in China, which is a multi-ethnic country and comprises diverse cultural systems.

As the national majority, the mainstream of Chinese culture is marked by Han Chinese culture, which emphasizes a balanced state of pleasant and unpleasant emotions (Luo et al., 2001). What appears to be happy (e.g., getting good grade in the exam) has its other side of the coin (e.g., invoking jealousy of others). Although happiness is desirable, it is necessary to control or at least reduce possible negative consequences (Leu et al., 2010). Indeed, there is now evidence suggesting that the experiences of mixed emotions are prevalent in Han Chinese culture (Spencer-Rodgers et al., 2010). It should be noted that as one of the biggest minorities in China, Mongolian Chinese have their own specific cultural values and characteristics (Oyunbileg et al., 2009). For example, Mongolian Chinese live relatively traditional lives, keep purer traditional Mongolian rituals, and celebrate Mongolian festivals (Yang et al., 2009). They have their own language (i.e., Mongolian), traditional activities (e.g., horse riding, wrestling), personality, and appearance. Rather than living in the crowded cities, Mongolian Chinese live in a spacious area (e.g., pastoral areas in Inner Mongolia). Such ecological and sociodemographic differences may shape and influence what they do and how they do it (Cai, 2011). Unlike Han Chinese who emphasize modesty and *Yin Yang* philosophy, Mongolian Chinese are considered to be bold, unconstrained, enthusiastic, and outgoing (Cai, 2011). Prior research also indicated that Mongolian Chinese are more extroverted than Han Chinese (Yang and Zhang, 2010). Also, ten characteristics of the Mongolians are identified, which are straightforwardness, tall stature, skilled wrestlers, nomadism, hospitality, good singers and dancers, simpleness, bravery, good drinkers, and diligence (Cai, 2011). The spacious living space and vigorous traditional activites may have great impact in shaping their unconstrained and outgoing characteristics and making their less endorsement on Confucian cultural values (Yang et al., 2009). Although Mongolian Chinese and Han Chinese share similar socially oriented values of collectivistic cultures (Oyunbileg et al., 2009), Mongolian Chinese are less subjected to the *Yin Yang* Philosophy and are more expressive emotionally (Li et al., 2012). Also, Han college students report higher levels of emotion regulation skills than Mongolian Chinese (Lu and Wang, 2012). Unlike Han Chinese who tend to control personal emotions in public, Mongolian Chinese have the traditions of celebrating festival together. They gather together to sing, dance, and express their happiness (Cai, 2011). Endorsement and daily practice in this cultural atmosphere may lead to the higher hedonism among Mongolian Chinese than Han Chinese (Lu and Wang, 2012). With this, it is possible that Han and Mongolian Chinese may experience different emotions in pleasant emotional events. Comparing Mongolian Chinese and Han Chinese will be a sensitive way to explore subcultural marks of experiencing mixed emotions.

### Implicit Assessment of Attitudes toward Happiness

Although previous studies have shown that explicit attitudes toward happiness may account for cultural differences in the experience of mixed emotions (Miyamoto et al., 2010), little research has been done to examine cultural differences in *implicit* attitudes toward happiness. There is, however, evidence showing that implicit and explicit attitudes toward happiness may play different roles on mixed emotions. For example, explicit attitudes toward happiness might have little effect on mixed emotions (Larsen and Mcgraw, 2011). In a different study using the Implicit Association Test (IAT) paradigm, participants who often implicitly evaluated happiness with pleasantness were less likely to report mixed emotions in their daily lives (Riediger et al., 2014). Thus, measuring implicit attitudes toward happiness contributes to complementing the growing literature of mixed emotions.

A meta-analysis on the relations between implicit measures and explicit measures suggested that explicit measures might have potential vulnerability in assessing personal and cultural beliefs. For example, people might report their beliefs in socially desirable ways in keeping with social norms (Hofmann et al., 2005). The validity of the implicit measures was considered to exceed that of explicit measures significantly, and were treated as a more robust predictor of personal and cultural beliefs (Greenwald et al., 2009). Also, implicit measures tend to be resistant to self-presentational motivations and social desirability concerns. As a typical implicit assessment of implicit attitudes toward happiness, IAT has been well validated and used to examine the implicit relative association strength between the target and attribute concepts (Walker and Schimmack, 2008; Riediger et al., 2014).

### The Present Study

In the present study, we aimed to investigate the effects of implicit attitudes toward happiness and contra-hedonic motivation on the mixed emotions in pleasant emotional events from a subcultural perspective (i.e., Han vs. Mongolian Chinese). Results from prior research indicated that contra-hedonic attitudes toward happiness and regulatory motivations are closely related to the changing philosophy of emotions which is promoted and dominated in Han Chinese culture (Spencer-Rodgers et al., 2010; Miyamoto et al., 2014). Because of the promoted cultural meaning system of emotions among Han Chinese, contra-hedonic attitudes toward happiness and regulatory motivations would be more predominant to make people experience mixed emotions in a pleasant emotional event. On the contrary, Mongolian Chinese are more hedonism than Han Chinese due to their own cultural values (Yang et al., 2009; Cai, 2011; Li et al., 2012). Their attitudes toward happiness and regulatory motivations are less influenced by mainstream Chinese culture (e.g., changing philosophy of emotions). Thus, the associations between contra-hedonic attitudes toward happiness, regulatory motivations, and mixed emotions would be attenuated among Mongolian Chinese. They may display different pattern of emotional experience in the pleasant emotional event because of their different habitual regulatory styles, cultural values, and personality characteristics. With this regard, differences in cultural values and emotion regulation imply the need to examine subculture (ethnicity) as moderators in the associations between attitudes toward happiness and regulatory motivation and mixed emotions.

Thus, in the present study, we used the IAT paradigm (Greenwald et al., 2003) to measure participants’ attitudes toward happiness, the Positive and Negative Affect Scale to measure participants’ mixed emotions, and a description task to measure contra-hedonic motivation.

There are two main reasons for positioning our research only on pleasant emotional events. First and foremost, previous research demonstrated that cultural differences between East Asians and Americans in mixed emotions were more representative in pleasant emotional events (Hui et al., 2009; Leu et al., 2010; Sims et al., 2015). Also, cultural differences are considered to be more salient in pleasant than in unpleasant emotions (Leu et al., 2011). Second, the current research focused on the influence of contra-hedonic attitudes toward happiness and contra-hedonic regulatory motivation in mixed emotions. It would be more sensitive to examine influences of contra-hedonic attitudes toward happiness and contra-hedonic regulatory motivation in a pleasant emotional event (Miyamoto et al., 2010). With these two considerations, in this study we only included pleasant emotional events but not unpleasant or neutral emotional events.

We investigated whether Han Chinese and Mongolian Chinese differ in the implicit attitudes toward happiness. In line with the claim that Chinese believe happiness can be potentially dangerous or obstructive and due to the domination of Han Chinese in Chinese culture (Miyamoto et al., 2014), we expected that Han Chinese would hold stronger contra-hedonic attitudes toward happiness in comparison to Mongolian Chinese (Hypothesis 1).

Second, modest correlations between attitudes toward emotions and mixed emotions are not surprising in light of previous studies documenting a moderating effect of culture in the association between attitudes toward happiness and mixed emotions. Thus, we examined the moderating effect of ethnic group in the associations between attitudes toward emotions and mixed emotions. We investigated how attitudes toward happiness relate to mixed emotions between Han and Mongolian Chinese. Based on the idea that Han Chinese are more likely to be influenced by the mainstream Chinese culture (Miyamoto et al., 2014), ethnic group might be an important factor which moderates the effect of attitudes toward happiness on mixed emotions. We expected Han Chinese to have a stronger association between contra-hedonic attitudes toward emotions and mixed emotions in a pleasant emotional event in comparison to Mongolian Chinese (Hypothesis 2).

Third, in accordance with the same idea of domination of Han Chinese in Chinese culture (Miyamoto et al., 2014), we expected that Han Chinese in comparison to Mongolian Chinese, would show stronger associations between contra-hedonic motivation and mixed emotions in a pleasant emotional event (Hypothesis 3).

## Materials and Methods

### Participants

Han Chinese undergraduates (*n* = 73, 61.6% female, *M*_age_ = 21.49 years, *SD* = 1.18 years) and Mongolian Chinese undergraduates (*n* = 46, 56.5% female, *M*_age_ = 20.65 years, *SD* = 1.45 years) from a public university in Shanghai volunteered to participate in this study. All participants were born in China. Both parents and grandparents of the Han Chinese were Han Chinese. All of the Mongolian Chinese participants in the present study self-identified as Mongolian Chinese. Both parents and grandparents of the Mongolian Chinese were Mongolian Chinese. All participants gave written consent to participate in the study.

### Materials and Procedure

The research protocol was approved by the Institutional Reviewing Board at Shenzhen University. Participants completed the study during two visits to the laboratory. Before data collection, all participants signed an informed consent. In the first evaluation session, participants completed a set of questionnaires regarding demographic information, experience of mixed emotions, and regulatory motivation (see below). One week later, they visited the laboratory again to finish the implicit measure of attitudes toward happiness.

#### Implicit Attitudes toward Happiness

In contrast to previous research (e.g., Riediger et al., 2014), in the current study we used the Single Category IAT (SC-IAT, Karpinski and Steinman, 2006) to measure participants’ implicit attitudes toward happiness. When using standard IAT, researchers need to have psychological contrasts between the two target categories and the two attribute categories (Zinkernagel et al., 2011). In many applications, psychological contrasts exist. For instance, “good” is the semantic contrast of “bad.” However, many targets and attributes do not have any psychological contrasts (Penke et al., 2006). In these situations, a comparative attitude or associative measure is not ideally suited for all research contexts.

Single Category Implicit Association Test is a modified version of the IAT that eliminates the need for the second contrast category (Zinkernagel et al., 2011). For example, SC-IAT has a target concept sharing a response key with positive evaluation in one of the blocks (e.g., happiness and positive versus negative), but with negative evaluation in the other (e.g., positive versus happiness and negative). It is proved to be reliable and valid to examine evaluative associations with a single attitude object in many social cognition domains (Siebler et al., 2010; Zinkernagel et al., 2011). Also, its implications in assessing implicit attitudes toward emotions is drawing attention recently (e.g., approach or avoidance attitudes toward fearful emotion, Hammer and Marsh, 2015).

Single Category Implicit Association Test can assess the evaluative associations with a single attitude object and provide a more specific measure of the evaluative associations in question than an standard IAT (Karpinski and Steinman, 2006). This task was used to examine the relative association strength between the targets (happiness) and the positive/negative attributes (good-bad). The task was administered with program Inquisit Software.

Participants categorized items according to three concepts by pressing different response keys. In the task, participants first saw a word at the center of the screen. Then they were asked to classify the word according to the indication of the concept to be emotionally pleasant, positive or negative as fast as possible by pressing the button “1” or “9.” An equal number of word items from each of these three semantic categories (i.e., happiness, positive, and negative) were presented. In the compatible block, participants were asked to sort word fitting happiness and positive concepts in the same key and negative concepts in a different key (20 practice and 40 test trials). In the incompatible block, participants were asked to sort word fitting happiness and negative concepts in the same key and positive concepts in a different key (20 practice and 40 test trials). Word stimuli of the SC-IAT were selected based on data from a prestudy in which 30 Chinese university students rated 30 words for the SC-IAT. Rating dimensions included familiarity and intensity. Based on the ratings of familiarity, we selected six words with the highest scores per category (e.g., happiness, positive, and negative). And we identified that the positive and negative stimuli lists did not differ significantly regarding their ratings of intensity. The items from the categories happiness, positive and negative were presented (**Table 1**). All of the items were presented in Chinese in the study.

**Table 1:** Items used in the Single Category Implicit Association Test (SC-IAT).

Happiness	Positive	Negative
Happiness	Luck	Poison
Joy	Beauty	Violence
Bliss	Gift	Sickness
Cheerfulness	Health	Failure
Merriment	Wisdom	Stench
Delight	Success	Stupidity

The shorter the reaction times the more strongly participants associate the two concepts (happiness-good or happiness-bad) sharing the same response key. *D*-values of the SC-IAT were calculated according to the D-Scoring algorithm proposed by Karpinski and Steinman (2006). Data from practices as well as test blocks were all included when calculating the *D*-values of the SC-IAT. Standard Deviations across practice and test trials were computed for each participant. Basic information about the SC-IAT was showed in **Table 2**. Average RTs for practice and test blocks were divided by the resulting standard deviations. Positive *D* scores indicate that participants implicitly considered happiness as positive (the hedonic attitudes toward happiness). Negative *D* scores indicate that participants implicitly considered happiness as negative (the contra-hedonic attitudes toward happiness). Higher *D* scores refer to more hedonic attitudes toward happiness.

**Table 2:** Basic information and differences comparisons about the happiness SC-IAT between two ethnic groups.

	*N* trials	Han Chinese (*N* = 73)	Mongolian Chinese (*N* = 45)	*t, Cohen’s d*
		**Mean (*SD)*** of RTs		
PC	20	925.71 (251.04)	772.77 (189.71)	3.51^∗∗∗^, 0.65
TC	40	801.77 (186.50)	712.17 (120.62)	2.87^∗∗∗^, 0.53
PI	20	643.97 (168.58)	611.14 (133.73)	1.11, 0.21
TI	40	591.33 (116.96)	607.21 (121.45)	-0.71, -0.13
		
		**Mean (*SD)* of *SD _pooled_***		
PB		470.68 (254.57)	382.01 (171.16)	2.06^∗^, 0.38
TB		363.73 (192.22)	346.77 (83.70)	0.56, 0.10

*PC, practice compatible block; TC, test compatible block; PI, practice incompatible block; TI, test incompatible block; PB, practice block; TB, test block.*

*^†^*p* < 0.10, ^∗^*p* < 0.05, ^∗∗^*p* < 0.01, ^∗∗∗^*p* < 0.001.*

#### Mixed Emotions

Participants were asked to write down a recent emotional event in which they felt happy and to indicate the degree to which they have experienced each of 12 emotions (adapted from the PANAS, Watson et al., 1988) in the pleasant emotional event by using a scale ranging from 1 (*not at all*) to 5 (*extremely*). The PANAS has proved to be reliable and valid in measuring emotion experiences among Chinese population (Miyamoto and Ma, 2011). We averaged ratings of energetic, excited, proud, and enthusiastic for pleasant emotional experiences (α*_HanChinese_* = 0.82, α*_Mongolian Chinese_* = 0.69) and ratings of sad, guilty, upset, nervous, fearful, ashamed, frighten, and anger for unpleasant emotional experiences (α*_HanChinese_* = 0.79, α*_MongolianChinese_* = 0.76). Mixed emotions were computed by using the negative acceleration model proposed by Spencer-Rodgers et al. (2010). The negative acceleration applied the formula ([2 × S] + 1)/(S + L + 2). For each individual, the participant’s larger mean emotional rating is categorized as the dominant response (L) and the smaller as the conflicting response (S). For example, if a participant’s mean pleasant emotion experience score is 5 and his or her mean unpleasant emotion experience score is 1, then pleasant emotion experience is the dominant response (or vice versa). Participants who feel predominantly pleasant or predominantly unpleasant emotions obtain relatively lower mixed emotion scores. Higher mixed emotion scores indicate greater co-occurrence of pleasant and unpleasant emotions.

#### Regulatory Motivation

After rating emotion experiences, participants also reported how they thought through the emotional event via open-ended questions. They were aksed, “*How did you think through this emotional event? Why did you do that?*” Regulatory motivations of the pleasant emotional events were coded into three categories: (a) hedonic motivation, (b) contra-hedonic motivation, and (c) no motivation. Regulatory motivations which were hedonic, need-oriented, and aimed to maximize favorable experiences were coded as hedonic motivation (e.g., “*I want to make myself feel happy,” “Because that make mee feel good”*). Regulatory motivations which were instrumental, goal-oriented and aimed to maximize utility of emotion experiences by dampening pleasant experiences or savoring unpleasant experiences were coded as contra-hedonic motivation (e.g., “*Modesty helps one go forward, whereas pride makes one fall behind,” “I want to get improve so I should not be so happy about my good grade*,” “*That (feeling good) is not the most important thing, we should focus on other things*”; Koole, 2009). No motivation was coded when participants reported they didn’t have any specific motives to their emotional experiences (e.g., “*I didn’t think anything when I felt happy at that moment*”). Research assistants completed extensive training about coding data prior to the start of the study. They coded a random subset of 36% of the responses (43 responses) to establish reliability. Interrater reliability between two raters was computed (Cohen’s *kappa* = 0.91). Raters met regularly during data coding to discuss any issues arising and to reduce rater drift. As to the average report rates of the three motivation categories, hedonic motivation was reported on average in 33.6% of cases and the contra-hedonic motivation was reported on average in 51.4% of cases. No motivation was reported on average in 15% of cases.

## Results

### Subcultural Difference in the Attitude Toward Happiness

We firstly compared the Happiness SC-IAT *D* scores with zero to confirm that whether happiness was evaluated differently by participants. SC-IAT D scores of both Han and Mongolian Chinese were significantly different from zero, *M_MongolianChinese_* = -0.38, *SD _MongolianChinese_* = 0.53, *t*(45) = -4.83, *p* < 0.001, 95% CI [-0.54, -0.22], *d* = -1.44; *M_HanChinese_* = -0.62, *SD_HanChinese_* = 0.23, *t*(72) = -23.29, *p* < 0.001, 95% CI [-0.67, -0.56], *d* = -5.49. To test subcultural differences in attitudes toward happiness, we compared *D* scores of the SC-IAT between Han and Mongolian Chinese. Results suggested that Mongolian Chinese held weaker contra-hedonic attitudes toward happiness than Han Chinese implicitly [*t*(117) = -3.39, *p* < 0.001, 95% CI [-0.38, -0.10], *d* = -0.63]. In comparison to Mongolian Chinese, Han Chinese evaluated happiness more negatively and implicitly. This result was consistent with our first hypothesis.

### Associations of Attitudes toward Happiness and Mixed Emotions in Different Subcultures

To test our second hypothesis, we firstly compared the self-reported mixed emotion score of Han Chinese with Mongolian Chinese. As expected, Han Chinese (*M_HanChinese_* = 0.83, *SD_HanChinese_* = 0.15) scored higher on the mixed emotions than did Mongolian Chinese (*M_MongolianChinese_* = 0.76, *SD_MongolianChinese_* = 0.14), *t*(117) = 2.48, *p* < 0.05, 95% CI [0.01, 0.12], *d* = 0.46.

When examining the subcultural differences in the correlation between pleasant and unpleasant emotions, we found that there was a strong positive correlation between pleasant and unpleasant emotions scores among Han Chinese (*r* = 0.65, *p* < 0.001), but no significant association was found among Mongolian Chinese (*r* = 0.11, *ns*). To examine the difference of the correlation between different groups, a Fisher’s r-to-Z transformation was used. Results showed that the correlations were significantly different from each other, *Z _diff_* = 3.34, *p* < 0.001.

**FIGURE 1:**
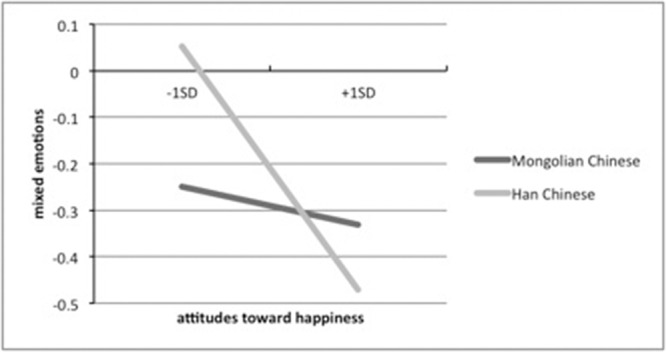
**Interaction between ethnic groups and attitudes toward happiness in predicting mixed emotions**.

A series of regression analyses were conducted to examine the relation between implicit attitudes toward happiness measured by SC-IAT and mixed emotions, as well as ethnic group as a moderating factor. Ethnic group was first entered into the equation to control for its effect in subsequent steps. Next, SC-IAT *D* score was entered into the equation. In the following step, interaction of the ethnic group and attitudes toward happiness was entered to examine the moderating effect. Variables were standardized to reduce multicollinearity in the analyses. Results indicated a significant main effect of ethnic group (β = -0.22, *p* < 0.05) whereby Han Chinese scored higher than Mongolian Chinese on mixed emotions. Implicit attitudes toward happiness was marginally associated with mixed emotions (β = -0.18, *p* = 0.055). In addition, there was significant interaction between ethnic group and attitudes toward happiness (β = 0.43, *p* < 0.05, also see **Table 3**). A simple slope test indicated that attitudes toward happiness negatively predict mixed emotions in Han Chinese (β = -0.31, *p* < 0.01) but not in Mongolian Chinese (β = -0.12, *ns*; **Figure 1**). In summary, in comparison to Mongolian Chinese, the more Han Chinese evaluated happiness as positive implicitly the less mixed emotions they experienced in a pleasant emotional event.

**Table 3:** Summary of regression analyses for ethnic group and attitudes toward happiness predicting mixed emotions.

Outcome: Mixed emotions	β	*t*	*R^2^*	*F*
Predictors				
Ethnic Group	-0.22^∗^	-2.48	0.11	4.78^∗∗^
Attitudes Toward Happiness	-0.18^†^	1.94		
Ethnic Group x Attitudes toward Happiness	0.43^∗^	2.00		

*^†^*p* < 0.10, ^∗^*p* < 0.05, ^∗∗^*p* < 0.01, ^∗∗∗^*p* < 0.001.*

### Associations of Regulatory Motivation and Mixed Emotions in Different Subcultures

To examine the differences in the frequency of reported hedonic and contra-hedonic motivation in the pleasant emotional event, cross-tabulation analysis was conducted. Results showed that the frequency of hedonic and contra-hedonic motivation of Han Chinese was not different than Mongolian Chinese in pleasant emotional events [χ^2^(1, *N* = 103) = 0.43, *p* = 0.51].

A series of regression analyses was conducted to examine the relation between regulatory motivation and mixed emotions, as well as ethnic group as a moderating factor. Ethnic group was first entered into the equation to control for its effect in subsequent steps. Next, regulatory motivation was entered into the equation. In the following step, the interaction of the ethnic group and regulatory motivation was entered to examine moderating effect. Results indicated a significant main effect of ethnic group (β = -0.64, *p* < 0.05) whereby Han Chinese scored higher than Mongolian Chinese on mixed emotions. Regulatory motivation was associated with mixed emotions (β = -0.27, *p* < 0.05). In addition, there was a marginally significant interaction between ethnic group and regulatory motivation (β = 0.54, *p* = 0.09, also see **Table 4**). A simple slope test indicated that regulatory motivation negatively predict mixed emotions in Han Chinese (β = -0.27, *p* < 0.05) but not in Mongolian Chinese (β = 0.07, *ns*).

**Table 4:** Summary of regression analyses for ethnic group and regulatory motivation predicting mixed emotions.

Outcome: Mixed emotions	β	*t*	*R^2^*	*F*
Predictors				
Ethnic group	-0.64^∗^	-2.16	0.07	2.57^†^
Regulatory motivation	-0.27^∗^	-2.13
Ethnic group x regulatory motivation	0.53^†^	1.71

*^†^*p* < 0.10, ^∗^*p* < 0.05, ^∗∗^*p* < 0.01, ^∗∗∗^*p* < 0.001.*

**FIGURE 2:**
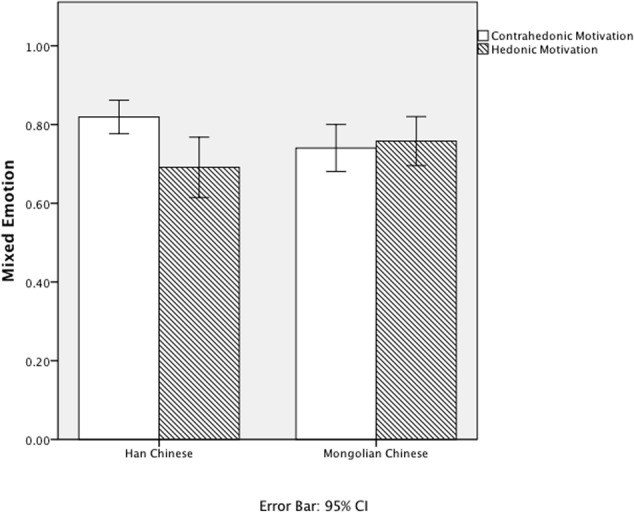
**Scores of the mixed emotions in different regulatory motivations between different subcultural groups**.

Then, we further examined whether Han and Mongolian Chinese differed in mixed emotions when they held different regulatory motivations during pleasant emotional events. We conducted *t*-tests to compare the scores of mixed emotions when participants had different regulatory motivations. Results showed that there were subcultural differences in the associations between regulatory motivation and mixed emotions (**Figure 2**). Among Han Chinese, participants who reported contra-hedonic motivation in the pleasant emotional event had a greater score of mixed emotions than those who had hedonic motivation, *t*(58) = 2.14, *p* < 0.05, 95% CI [0.005, 0.15], *d* = 0.56. No significant differences were found in mixed emotions between who had contra-hedonic and hedonic motivation among Mongolian Chinese [*t*(41) = -0.42, *p* = 0.68, *d* = 0.13]. Results were consistent with our third hypothesis.

## Discussion

Prior studies suggested that mixed emotions are more prevalent in Chinese than in Americans (Goetz et al., 2008; Spencer-Rodgers et al., 2010). However, as a multi-ethnic country, the dominant Han Chinese culture cannot fully represent the whole Chinese population. Our research was motivated in our interest to understand why people experience mixed emotions at the same time in a pleasant emotional event among subcultural groups. In this study, we wanted to know how implicit attitudes toward happiness and contra-hedonic regulatory motivation influence the mixed emotions in different subcultural groups in China.

Pleasant emotions are not always considered to be “positive” across cultures. Prior research indicated that pleasant emotions may not be “positive” for Asians because of cultural differences in the meaning assigned to pleasant emotions (Leu et al., 2011). In line with findings from other studies (Uchida and Kitayama, 2009; Miyamoto et al., 2010), our findings first revealed contra-hedonic attitudes toward happiness in both Han Chinese and Mongolian Chinese. Unlike Western counterparts, both Han Chinese and Mongolian Chinese implicitly linked happiness with negative concepts more strongly than positive concepts. Moreover, Mongolian Chinese held weaker contra-hedonic attitudes toward happiness than Han Chinese. Moderation analysis suggested that ethnic group moderated the effects of attitudes toward happiness on the experience of mixed emotions. Subcultural differences in attitudes toward happiness were largely corresponded to the extent to which people are shadowed by mainstream Chinese culture. Specifically, attitudes toward happiness are dialectical and marked by a changing philosophy (Luo et al., 2001). Given that pleasant experiences from pleasant emotional event have embedded potential negative consequences for the future (Tsai et al., 2006), mixed emotions in a pleasant emotional event reflect a compromising balance between a desirable hedonic experience and the potential negative impact of pleasant emotions. Such contra-hedonic attitudes toward happiness are more prevalent in Han Chinese (Miyamoto et al., 2014). Thus, subcultural differences in mixed emotions could be attributed to subcultural differences in contra-hedonic attitudes toward happiness.

When reporting pleasant emotional events, both Han Chinese and Mongolian Chinese exhibited contra-hedonic regulatory motivation. Rather than fulfilling one’s hedonic needs, they intended to enhance unpleasant emotions or reduce pleasant emotions by utilitarian considerations (Tamir et al., 2007). This result was consistent with previous evidence that contra-hedonic motivation are more likely to keep people’s emotional responses in a relatively safe range and reduce the potential negative consequences of strong pleasant emotions (Riediger et al., 2014). Although Han Chinese did not differ in the frequency of hedonic and contra-hedonic motivation from Mongolian Chinese in terms of pleasant emotional events, subcultural differences in the relations between regulatory motivation and mixed emotions were found. Specifically, Han Chinese with contra-hedonic motivation in the pleasant emotional event experienced higher level of mixed emotions than those who had hedonic motivation, but these differences were not found in the Mongolian Chinese. Such subcultural differences may derive from the notions that contra-hedonic motivation is more prevalent in Han Chinese than in Mongolian Chinese (Miyamoto and Ma, 2011). Therefore, contra-hedonic motivation may play a more important role in influencing mixed emotions in Han Chinese. This interpretation supports that Chinese who held contra-hedonic regulatory motivation would tend to experience more mixed emotions in daily lives (Goetz et al., 2008). The stronger association between contra-hedonic motivation and the mixed emotions in Han Chinese strengthens the assumption that the prevalence of mixed emotions differ depending on subcultural background. In the pleasant emotional event, the mixed emotions may help people to prevent experiencing extreme pleasant emotions. Thus, keeping balance between pleasant and unpleasant emotions may avoid the potential risk from pleasant emotion, in turn adapts the core regulation goal under Chinese background – maintaining interpersonal harmony (Uchida and Kitayama, 2009). A developmental study suggested that Chinese adolescents who habitually down-regulate their pleasant emotions would have more adaptive emotional outcomes during development (Sang et al., 2014). Therefore, simply emphasizing the elevation of pleasant emotions fails to account for the adaptive nature of emotions. It is important for researchers to understand that the co-occurrence of pleasant and unpleasant emotions occur–mixed emotions might play an important role in influencing individuals’ emotional lives under different cultural contexts.

It is interesting to note that our results supported subcultural differences in attitude toward happiness but not regulatory motivation. Frequency of hedonic and contra-hedonic motivation of Han Chinese was not different from Mongolian Chinese in the pleasant emotional events. Although there were subcultural difference in attitude toward happiness, our findings indicated that both groups implicitly considered happiness as negative. They both held the contra-hedonic attitudes toward happiness despite varied in degree. As attitudes toward emotions influence what people prefer to experience (Larsen and Mcgraw, 2011), the contra-hedonic attitudes toward happiness might lead to a contra-hedonic regulatory motivation in the reported pleasant emotional events. On the other hand, methodologically, we measured attitude toward happiness and regulatory motivation via different methods. Subcultural difference in implicit attitude toward happiness was assessed by an IAT paradigm. It was resistant to self-presentational motivations and social desirability concerns (Greenwald et al., 2009). Regulatory motivation was self-reported. Thus, this might explain why the subcultural difference in regulatory motivation was absent. Moreover, as stated, attitudes toward happiness are kind of evaluations and representations toward happiness emotions (Riediger et al., 2014). It focuses on the emotions themselves. Unlikely, regulatory motivation is a behavioral and regulatory tendency in a specific emotional event (Miyamoto et al., 2014) that underlines the emotional responses. They might imply different domains of individuals’ emotional preferences. It is important to examine the underlying relations between such two constructs in future studies.

### Limitations and Future Directions

Our findings further demonstrated that there are not only cross-cultural differences – there are also subcultural differences in mixed emotions. Specifically, Han Chinese showed higher mixed emotions than did Mongolian Chinese in the pleasant emotional event. However, the data in this study were cross-sectional and do not allow a casual test of the relations between mixed emotions, attitudes toward happiness, and regulatory motivation. Thus, it is still unclear whether findings can be generalized to situations where attitudes toward happiness and regulatory motivation are induced by using experimental manipulations. Future studies should examine the effects of different subcultural values on the attitudes toward happiness, regulatory motivation, and mixed emotions, and implement experimental assessments that allow evoking on-line regulatory motivation and attitudes toward happiness. Also, measurement of emotion experience (PANAS) and regulatory motivation (open-ended response) were self-report based, and is therefore prone to retrospective and self-presentation biases. Thus, it will be important to examine emotion experience through objective measurements (e.g., physiological and neural changes).

There is also an important point which ought to be considered when interpreting the present results. The operationalization of “happiness” influence how people evaluate (attitude toward happiness) and want to experience (regulatory motivation) in a pleasant emotional event (Leu et al., 2010). For example, prior work has shown that low-arousal positive affects (e.g., calm and peaceful) are considered as safer and more desirable pleasant emotional states in Chinese culture. However, extreme pleasant emotions beget negative consequences (Miyamoto et al., 2014). According to the Affect Valuation Theory (Tsai et al., 2006), pleasant emotions examined as “happiness” in our study were skewed toward high arousal positive affect (e.g., the words used for the “happiness” category in the SC-IAT and the PANAS positive emotion words). It is unclear whether a low-arousal definition of happiness may influence our findings of subcultural differences. Thus, it will be fruitful for future work to examine this same research question with a low-arousal definition of happiness.

Given that all the participants of our study were adults, we did not include measures about parents’ educational level, occupation, or socio-economic status. However, social classes may create variability in individuals’ social lives and psychological orientations. With this, it will be interesting to include measures of social classes and explore their underlying relations with mixed emotions in future studies. Moreover, it will be interesting to examine the differences in mixed emotions between the Chinese Americans who are born, raised and more exposed to hedonist culture versus Chinese from China where the mainstream culture is contra-hedonic. Also, findings from the present study provided some potential implications to the intervention of affective disorders. Even within a culture, Mongolian Chinese and Han Chinese showed attitudes toward emotions, motivations in emotion regulation, and emotion experience differently depending on subculture. These subcultural differences have significant influence on how people perceive, monitor, and regulate their emotion experience when they are in a vulnerable condition. Thus, subcultural diversity in individuals’ emotional lives exists and is worthy of attention. Not only researchers in the field of emotions but also psychiatrists need to accommodate subcultural diversity when designing interventions for affective disorders targeted at different subcultural groups.

## Conclusion

This study demonstrated that subculture (ethnicity) moderated the associations between attitudes toward happiness and regulatory motivation and mixed emotions. Together with other studies that show cross-cultural differences in mixed emotions, findings from this study shed light on subcultural differences in mixed emotions under a diverse cultural background.

## Author Contributions

XD is the first and corresponding author of this paper. She made contributions to conception and design of the study, acquisition of data, analysis on data, interpretation of data, drafting the article, and finalizing the article. XD mainly contributed to the study by analyzing data and helping to finalize the article. CC mainly contributed to the study by helping to draft and finalize the article. HC mainly contributed to the study by helping to finalize the article and do proofreading.

## Conflict of Interest Statement

The authors declare that the research was conducted in the absence of any commercial or financial relationships that could be construed as a potential conflict of interest.
